# Giant cell angiofibroma of gingiva in tuberous sclerosis complex: a case report and literature review

**DOI:** 10.1186/s13000-024-01467-4

**Published:** 2024-03-08

**Authors:** Qiaochu Sun, Jaeyoung Ryu, Young Kim

**Affiliations:** 1 School of Stomatology, Dalian Medical University, Dalian, 116044 China; 2 Department of Oral and Maxillofacial Surgery, School of Dentistry, Chonnam National University, 77 Yongbong-ro, Buk-gu, Gwangju, 61186 Republic of Korea; 3 Department of Oral Pathology, School of Dentistry, Chonnam National University, 77 Yongbong-ro, Buk-gu, Gwangju, 61186 Republic of Korea

**Keywords:** Tuberous sclerosis, Giant cell angiofibroma, Gingival enlargement

## Abstract

**Background:**

Tuberous sclerosis complex (TSC) is a rare, complex genetic disorder characterized by hamartomas and neoplastic lesions in various organ systems. With the development of radiology and gene testing, the diagnostic criteria for TSC were updated in 2012 at the International Consensus Conference. Intraoral fibromas have long been associated with TSC. However, the incidence of giant cell angiofibroma (GCA) in TSC patients is extremely rare. Here, we report the first case of GCA in the gingival tissue of a patient with TSC.

**Case presentation:**

A 41-year-old woman first visited the Department of Oral and Maxillofacial Surgery, Chonnam National University Dental Hospital, complaining of gingival enlargement. Clinical examination revealed several manifestations associated with TSC, including intraoral fibromas, facial angiofibromas, dental enamel pits, ungual fibromas, “confetti” skin lesions, hypomelanotic macules, and a shagreen patch. Intraoral examination revealed a 6.0 × 5.0 cm gingival overgrowth on the left mandible. Surgical excision was performed, and subsequent histopathological examination confirmed the diagnosis of GCA. There was no evidence of recurrence within the 24- months of surgery.

**Conclusions:**

We report the first case of GCA in the gingival tissue of a patient with TSC. This report would contribute to an improved understanding of this rare disease. However, further case reports are necessary to clarify the relationship between GCA and TSC.

## Background

Tuberous sclerosis complex (TSC) is a rare, multisystem, autosomal dominant neurocutaneous disorder that affects multiple sites of the body [1]. TSC was first described approximately 160 years ago by German pathologist Friedrich Daniel von Recklinghausen in 1862 [2]. The most common findings of TSC are hamartomas in the skin, central nervous system, kidney, lung, heart, retina, gingiva, and other abdominal organs [3].

TSC invading the brain is associated with various neurological symptoms, including epilepsy, mental retardation/developmental delay, and psychiatric illness [1]. Medical problems affecting the central nervous system are the leading cause of morbidity and mortality in TSC. Renal manifestations of TSC commonly include angiomyolipomas, cysts, and renal cell carcinomas. In the cardiac context, TSC often presents as rhabdomyomas and arrhythmias. Pulmonary features most common in TSC are lymphangioleiomyomatosis [4] and multifocal micronodular pneumocyte hyperplasia [5]. Most patients affected by TSC exhibit dermatological or dental findings. Skin manifestations associated with TSC include hypomelanotic macules, “confetti” skin lesions, facial angiofibromas, fibrous cephalic plaques, shagreen patches, and ungual fibromas [3]. Among oral lesions, dental enamel pits are more prevalent in TSC patients than in the general population, and intraoral fibromas can be found on the gingiva, buccal or labial mucosa, and tongue [1]. Intraoral fibromas have long been associated with TSC, with gingival fibromas occurring in approximately 20–50% of TSC patients [6–8]. However, giant cell angiofibromas in patients with TSC are extremely rare. In this case report, we present the first giant cell angiofibroma in the gingival tissue of a patient with TSC.

## Case presentation

**Fig. 1:**
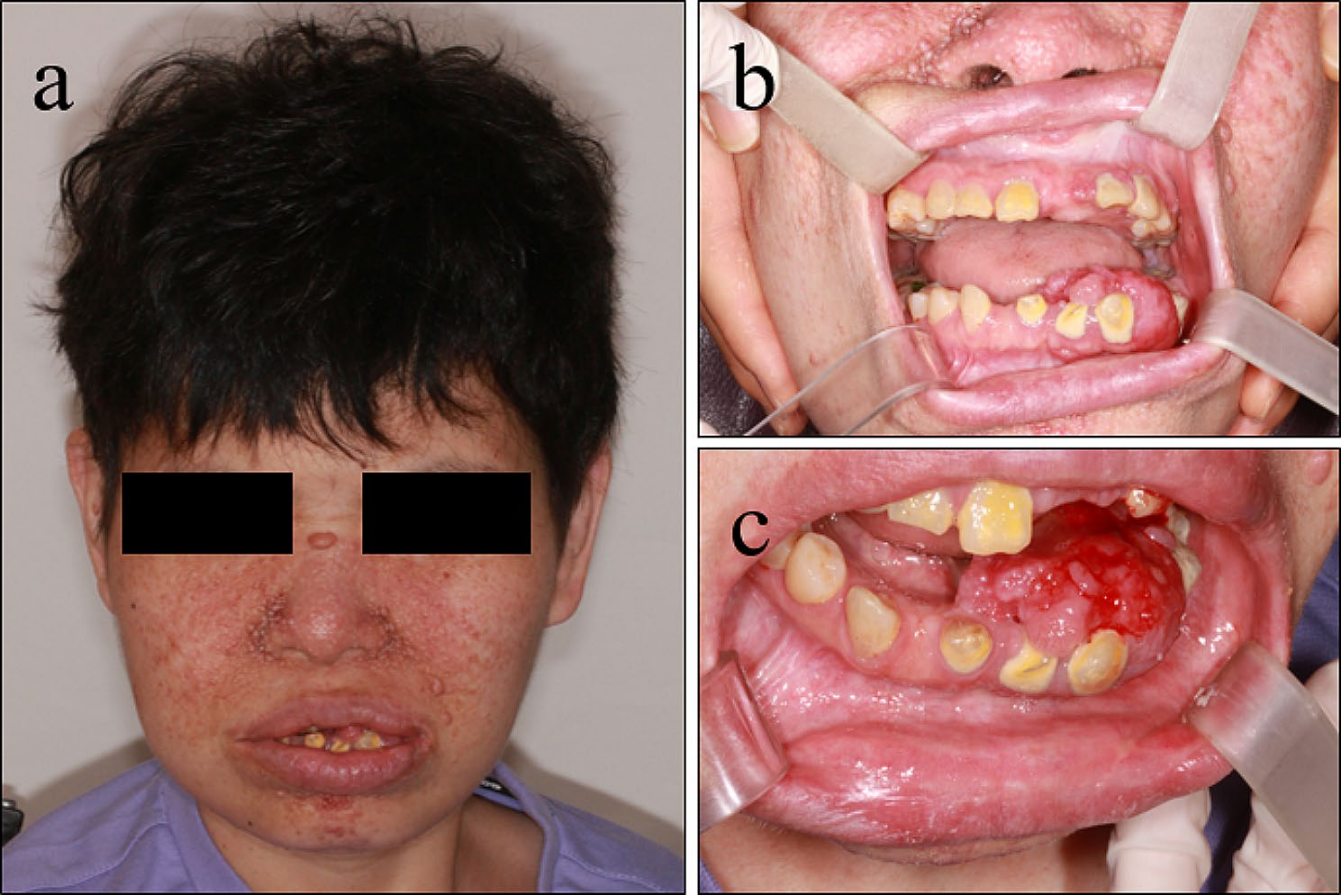
Tuberous sclerosis complex. **a** Multiple confluent angiofibromas involving the malar region, nose, cheeks, and chin. **b-c** Gingival enlargement on the left mandibular region and bleeding on the enlarged gingival surface

**Fig. 2:**
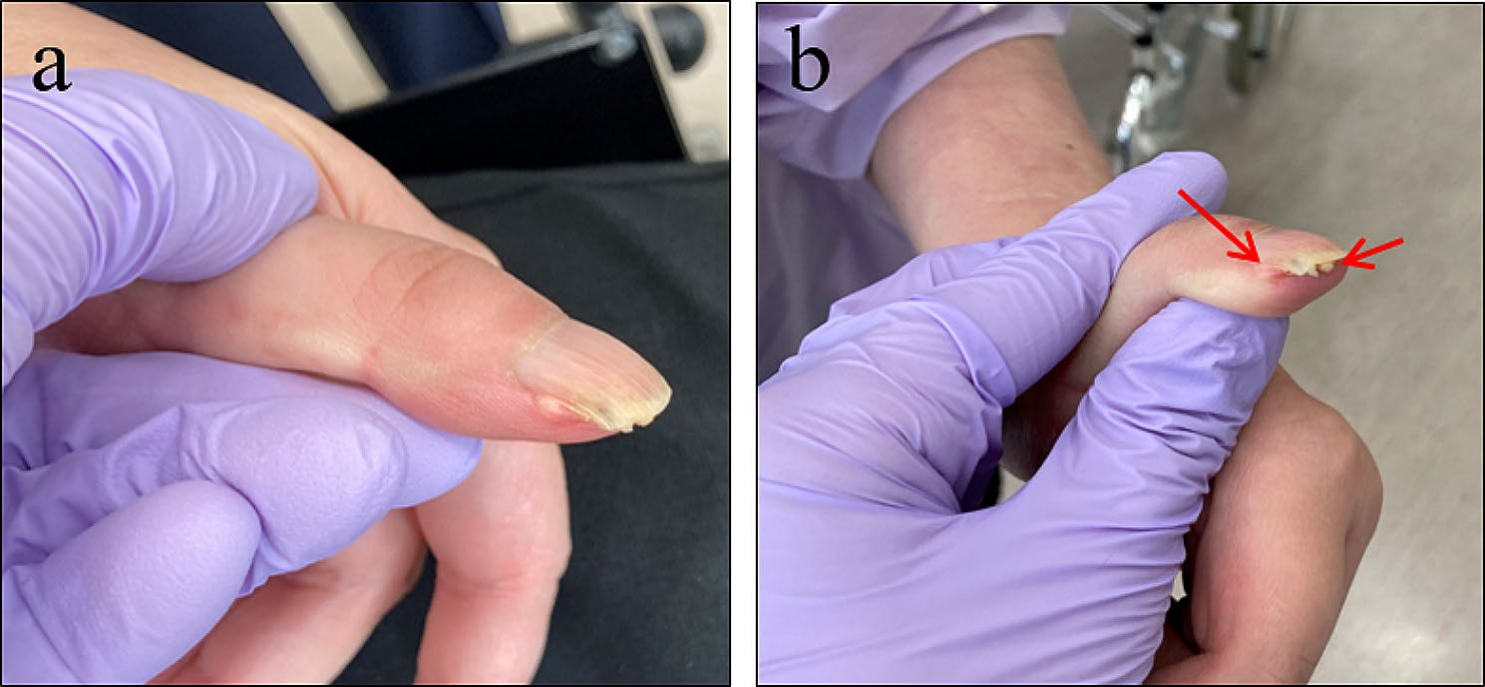
Ungual fibromas (indicated by arrows)

**Fig. 3:**
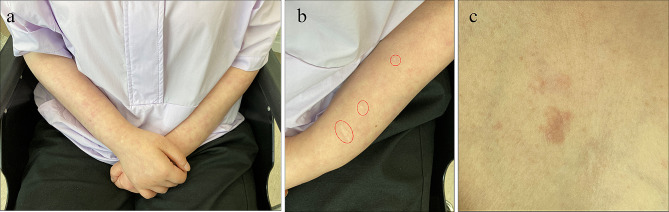
Cutaneous manifestations. **a** “Confetti” skin lesions on arms. **b** Three hypomelanotic macules on the left arm (circled area). **c** Shagreen patches on the chest

A 41-year-old woman first visited the Department of Oral and Maxillofacial Surgery, Chonnam National University Dental Hospital, in June 2021 for the excision of a gingival overgrowth in the left mandible area. The patient was diagnosed with tuberous sclerosis complex (TSC) at birth. She received treatment for epilepsy for 20 years, managed hypothyroidism for 8 years, and underwent angioembolization surgery for acute pyelonephritis in 2014. The patient had impaired vision, mental retardation, and an inability to communicate. Notably, she had conspicuous cutaneous nodules on the lower 2/3 of her face (Figure 1a), which remained relatively unchanged for several decades. However, she recently developed prominent and progressively increasing gingival overgrowths over the past two to three years. Intraoral examination revealed a 6.0 × 5.0 cm gingival overgrowth in the left mandibular region, extending from the mandibular left central incisor to the left first molar (Fig. 1b-c). Given the patient’s history of long-term epilepsy medication use (sodium valproate, gabapentin, lamotrigine), the enlarged gingival lesion was suggestive of drug-induced gingival hyperplasia. Multiple enamel pitting (>3) was present throughout the dentition, with the overlying gingiva displaying a loss of stippling. The patient maintained good oral hygiene, with no evident periodontitis or caries. Due to the patient’s inability to cooperate, radiographic surveys (panoramic imaging or cone beam computed tomography) were unavailable. Additionally, two ungual fibromas were observed on the patient’s left thumb, appearing over the lateral nail groove (peri-ungual) and beneath the nail plate (subungual) (Fig. 2). The patient also displayed scattered “confetti” skin lesions on both arms (Fig. 3a) and three hypomelanotic macules on the left arm (Fig. 3b). Furthermore, a shagreen patch with an uneven/bumpy surface was observed on the chest (Fig. 3c).

**Fig. 4:**
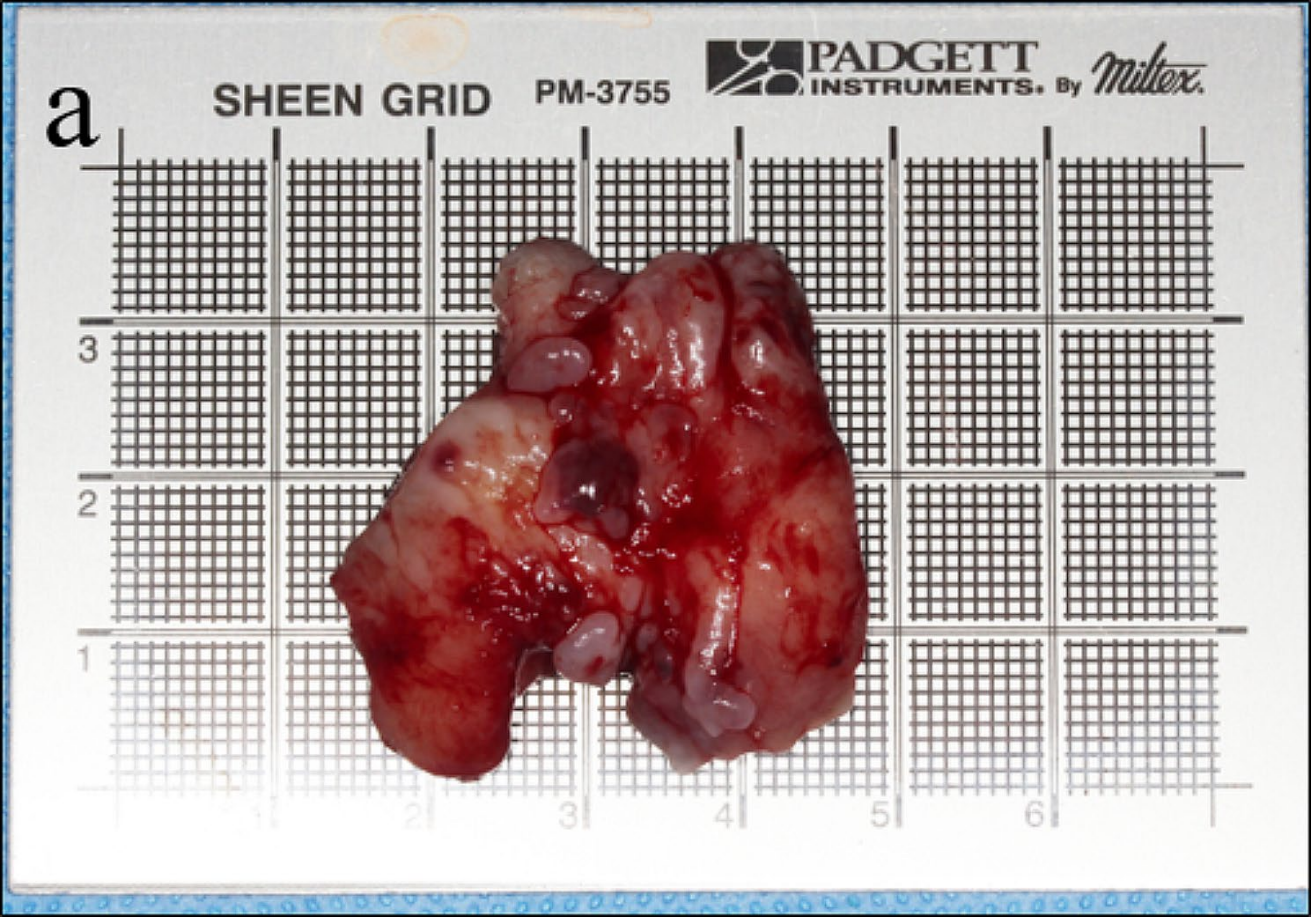
Appearance of the surgically excised gingival mass. The mass was 3.5 × 3.5 cm, irregularly shaped with a pink to deep red color

**Fig. 5:**
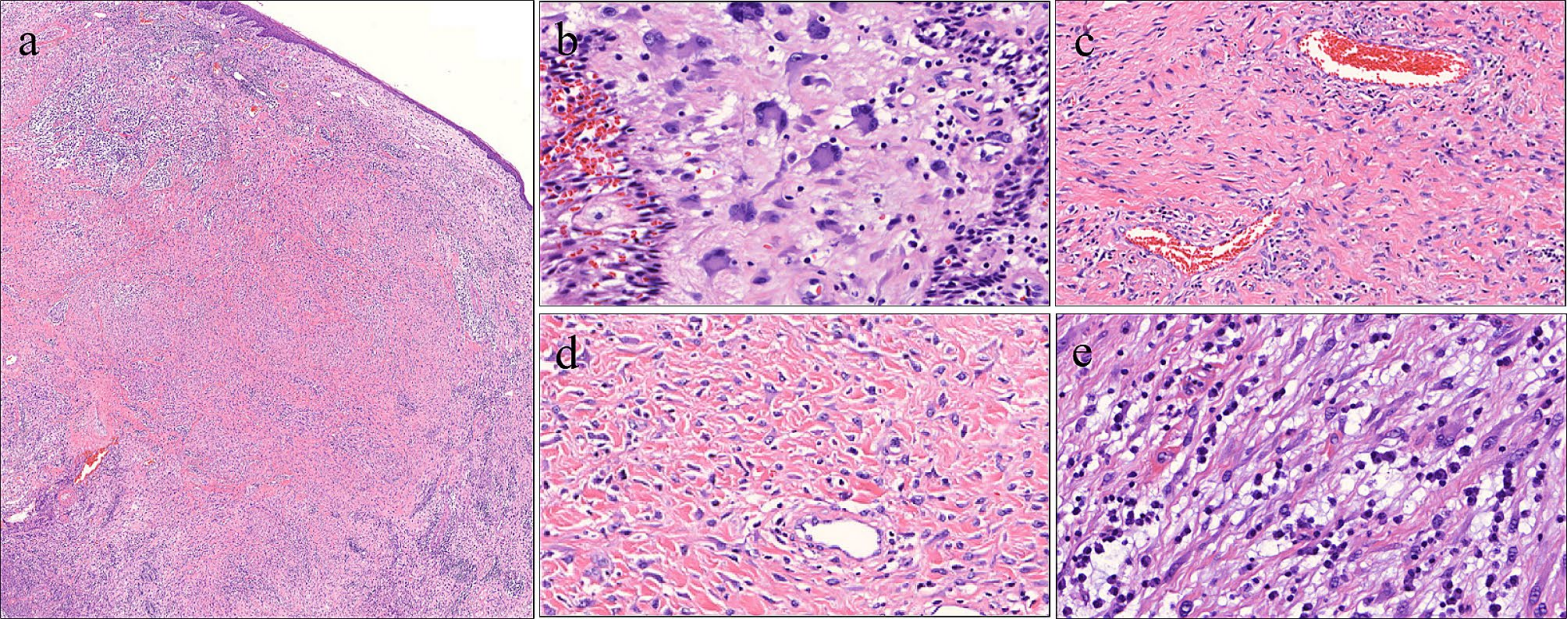
Microscopic findings of the gingival mass in the tuberous sclerosis complex (TSC) patient. **a** Low-power magnification view showing the tumor covered with hyperplastic squamous epithelium (hematoxylin-eosin staining; magnification, 10×). **b** Higher magnification showing a wreathlike arrangement of nuclei in interspersed cells, resembling Touton giant cells (hematoxylin-eosin staining; magnification, 200×). **c** Ectatic blood vessels surrounded by proliferating tumor cells (hematoxylin-eosin staining; magnification, 100×). **d** Numerous pleomorphic, stellate-shaped, and plump fibroblasts (hematoxylin-eosin staining; magnification, 200×). **e** Typical plasma cells around mass (hematoxylin-eosin staining; magnification, 200×)

Following a comprehensive extra-oral and intra-oral examination, surgical excision was planned for the patient, with appropriate informed consent obtained from the guardian. The procedure was performed under general anesthesia to remove the enlarged gingival tissue in the left mandibular segment. The excised tissue was submitted for histologic examination. Macroscopically, the mass was 3.5 × 3.5 cm and showed an irregular shape with a pink to deep red color (Fig. 4). Subsequently, the tissue was fixed in 10% formalin, embedded in paraffin, and stained with hematoxylin-eosin. Histologically, the section exhibited a tumor covered with keratotic squamous epithelial lining approximately 15–20 cells thick (Fig. 5a). Within the underlying tissue, Touton giant cells with nuclei arranged in a wreathlike pattern were interspersed (Fig. 5b, contained), along with numerous ectatic blood vessels (Fig. 5c). There were proliferating pleomorphic, stellate-shaped, multinucleated fibroblasts (Fig. 5d) and typical plasma cells aggregated around the lesion (Fig. 5e).

**Fig. 6:**
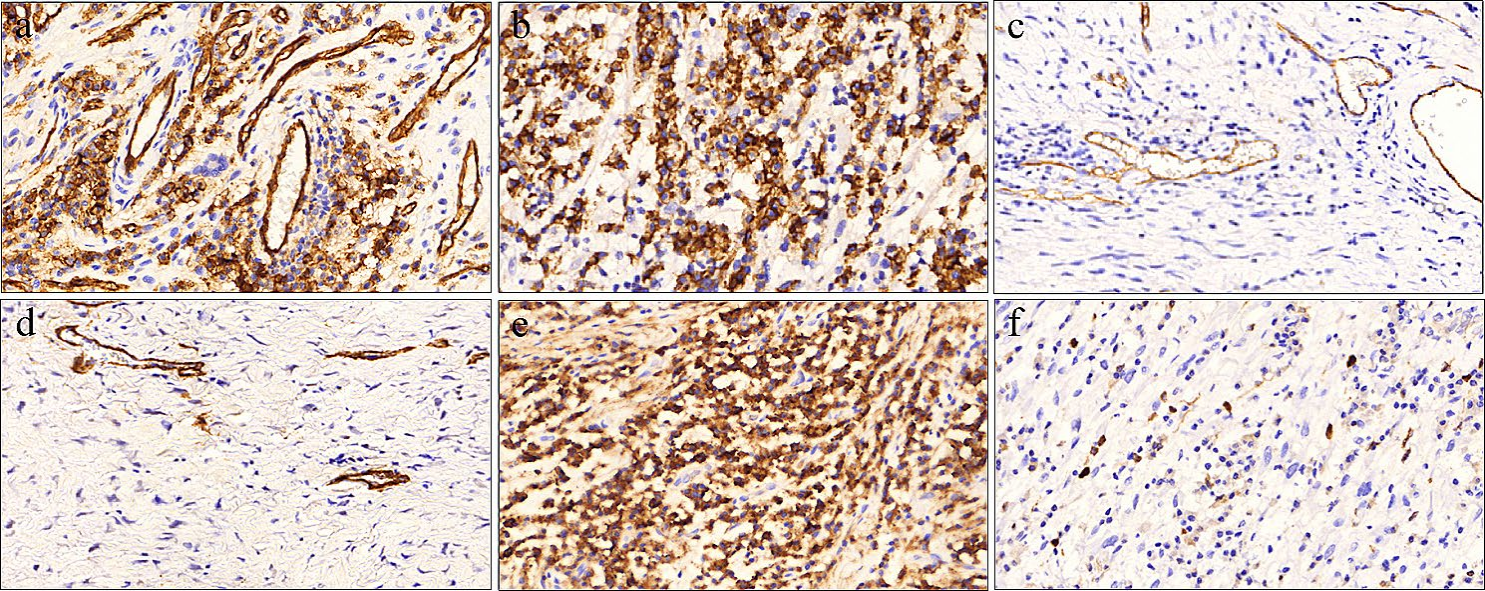
Immunohistochemical findings of gingival giant cell angiofibroma in the TSC patient. **a-b** Immunostaining for CD34 (**a**) and Bcl-2 (**b**) in tumor cells showed positive results (magnification, 200×). **c-d** CD31 (**c**) and SMA (**d**) were negative for tumor cells. **e-f** CD138 was positive for plasma cells around tumor and Ki67 labeling index was 1–2% (magnification, 200×)

**Fig. 7:**
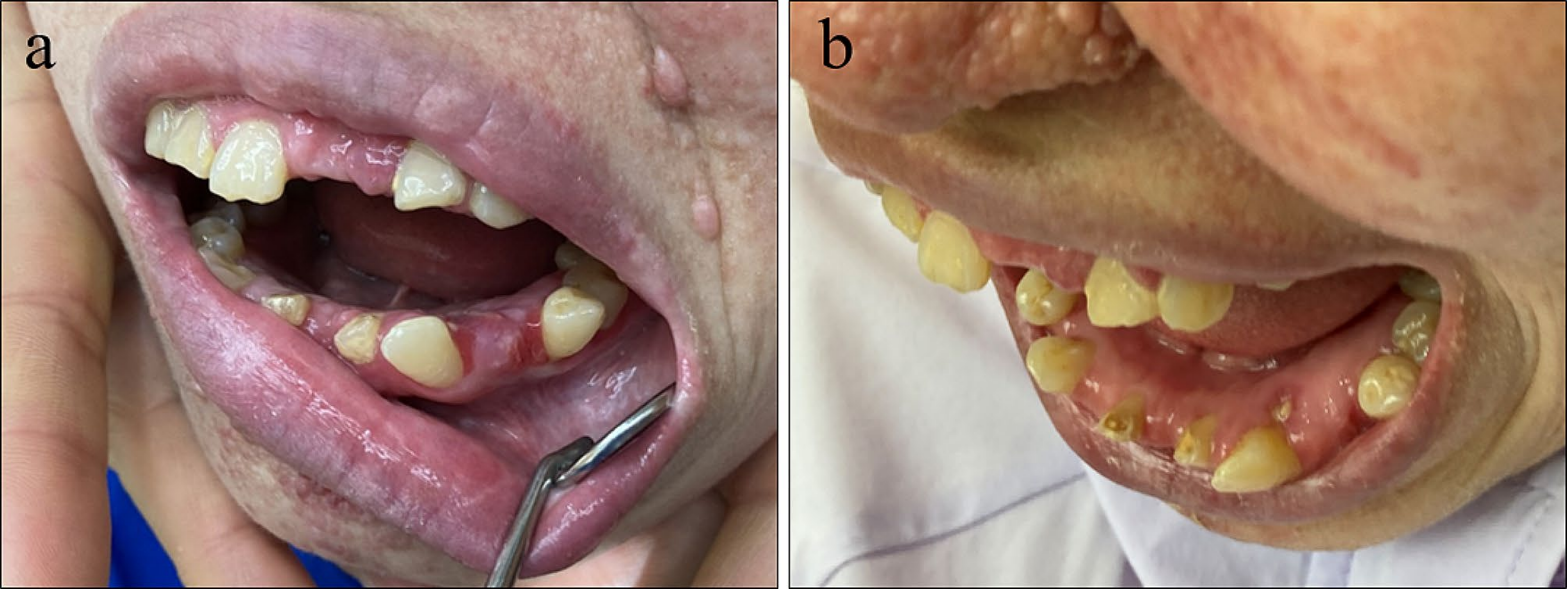
**a** 3-month postsurgical recall. **b** 1-year postsurgical recall

Immunohistochemistry analysis revealed distinct expression of CD34 and Bcl-2 in the tumor cells, which is compatible with the findings of GCA (Fig. 6a-b). Expression of CD31 and smooth muscle actin (SMA) were positive in the vessel area within the tumor but not in the tumor cells (Fig. 6c-d). CD138 showed positive expression in the plasma cells surrounding the tumor and the Ki67 index of the tumor was determined as 1–2% (Fig. 6e-f). Based on these findings, the pathological diagnosis confirmed the presence of giant cell angiofibroma (GCA) in the gingiva of the TSC patient. During the follow-up visit at 3 months (Fig. 7a) and 1 year (Fig. 7b) post-surgery, the gingival surface and contours displayed uneventful healing, with no indications of recurrence.

## Discussion and conclusions

TSC is a rare, complex genetic disorder with variable manifestations. The diagnostic criteria established in 1998 were revised in the 2012 International TSC Consensus Conference to include genetic testing as an independent diagnostic criterion [1]. Moreover, the conference reduced diagnostic classes from three (possible, probable, and definite) to two (possible, definite). The updated clinical diagnostic criteria are based on the presence of 11 major features and 6 minor features [1]. The major features include (1) hypomelanotic macules (≥ 3, at least 5-mm diameter), (2) angiofibromas (≥ 3) or fibrous cephalic plaque, (3) ungual fibromas (≥ 2), (4) shagreen patch, (5) multiple retinal hamartomas, (6) cortical dysplasias, (7) subependymal nodules, (8) subependymal giant cell astrocytoma, (9) cardiac rhabdomyoma, (10) lymphangioleiomyomatosis (LAM), (11) angiomyolipomas (≥ 2). The revised minor features include (1) “confetti” skin lesions, (2) dental enamel pits (> 3), (3) intraoral fibromas (≥ 2), (4) retinal achromic patch, (5) multiple renal cysts, (6) nonrenal hamartomas (Table 1).

**Table 1:** Updated clinical diagnostic criteria for tuberous sclerosis complex

Clinical diagnostic criteria	Clinical findings	This case
Major features	1. Hypomelanotic macules (≥ 3, at least 5-mm diameter)	+
	2. Angiofibromas (≥ 3) or fibrous cephalic plaque	+
	3. Ungual fibromas (≥ 2)	+
	4. Shagreen patch	+
	5. Multiple retinal hamartomas	-
	6. Cortical dysplasias*	-
	7. Subependymal nodules	-
	8. Subependymal giant cell astrocytoma	-
	9. Cardiac rhabdomyoma	-
	10. Lymphangioleiomyomatosis (LAM)†	-
	11. Angiomyolipomas (≥ 2)†	-
Minor features	1. “Confetti” skin lesions	+
	2. Dental enamel pits (> 3)	+
	3. Intraoral fibromas (≥ 2)	+
	4. Retinal achromic patch	-
	5. Multiple renal cysts	-
	6. Nonrenal hamartomas	+

Definite diagnosis: Two major features or one major feature with ≥ 2 minor features

Possible diagnosis: One major feature or ≥ 2 minor features

*Includes tubers and cerebral white matter radial migration lines

†A combination of the two major clinical features (LAM and angiomyolipomas) without other features does not meet the criteria for a definite diagnosis

A definite diagnosis of TSC is based on the following criteria: two major features or one major feature with two or more minor features. A possible diagnosis, however, is based on either one clinical major feature or two (or more) minor features. Additionally, the presence of a heterozygous pathogenic variant in TSC1 or TSC2 identified through molecular genetic testing is considered an independent genetic diagnostic criterion for TSC [1]. In the present patient, a definite diagnosis of TSC was based on the observation of four major features (hypomelanotic macules, angiofibroma, ungual fibromas, and shagreen patches) and three minor features (“confetti” skin lesions, intraoral fibromas, and dental enamel pits (> 3)).

Within the diagnostic criteria for TSC, intraoral fibromas and dental pitting are recognized as two minor features [1]. Intraoral fibromas may be present on the gingiva [1], buccal or labial mucosa, and even the tongue in TSC patients [8]. Sparling, J.D., et al. documented the oral clinical manifestations of 58 TSC patients and classified intraoral fibromas based on their location, either gingival or non-gingival [8]. According to their findings, gingival fibromas were observed in 30 patients (52%), while fibromas in other oral sites were found in 23 patients (40%). The non-gingival sites affected by intraoral fibromas in TSC patients included the buccal mucosa (24%), labial mucosa (17%), superior labial frenulum (9%), hard palate (3%), and tongue (3%) [8]. According to the clinical diagnostic criteria for tuberous sclerosis complex, the patient’s gingival mass on oral physical examination was first thought to be an intraoral fibroma. However, the mass was later confirmed to be GCA through pathological findings of the surgical specimen.

The enlarged gingiva of TSC patients should be differentiated from inflammatory, familial, or medication-induced gingival enlargement. In the present case, the patient maintained adequate oral hygiene, and the observed gingival manifestation did not align with the characteristics associated with gingivitis. Furthermore, there was no familial history of gingival enlargement, and histological examination revealed the absence of elongated, narrow rete ridges of epithelium typically observed in familial-induced gingival enlargement [9]. Therefore, inflammation and familial history were ruled out as causes of gingival enlargement. Gingival enlargement is also caused by medications, such as phenytoin (an anti-epileptic), calcium channel blockers, or cyclosporine [10]. Though the patient’s medication history of anti-epileptic drugs (sodium valproate, gabapentin, and lamotrigine) suggested drug-induced gingival overgrowth, there were no microscopic characteristics, such as excessive collagen deposition, with no fibroblastic proliferation [10].

The literature review on TSC commonly describes gingival enlargement as non-specific gingival enlargement [11–16]. However, unlike other case reports with usual fibromas, histological evaluation of the excised gingival tissue, in this case, revealed multiple Touton-type giant cells, numerous dilated capillaries, and abundant pleomorphic, stellate-shaped fibroblasts consistent with giant cell angiofibroma. Kacerovska, D., et al. [17] found Touton giant cells in cutaneous specimens of a TSC patient and established a diagnosis of GCA based on histological examination. In their report, the authors used 42 lesional specimens from large disfiguring facial angiofibromas in a patient with TSC. Based on the histopathologic results, localized lymphedema could contribute to the pathogenesis and appearance of GCA in TSC.

GCA was first reported as an orbital tumor by Dei Tos et al. in 1995 and is known to be a tumor in the orbital region [18]. It is rare for GCA to occur in extraorbital locations, including axillary-inguinal regions, posterior mediastinum, head and neck, hip, vulva, nasolacrimal canal, and oral cavity [19]. Although GCA is an independent lesion, reports show an association with an eyelid mass in a TSC patient and a cutaneous lesion in the thigh of a patient with dermatofibrosarcoma protuberans [20]. Histopathologically, GCA shows proliferated patternless spindle cells, floret-like multinucleated giant cells, and pseudovascular spaces [21]. Due to its pathological similarities with solitary fibrous tumor (SFT), GCA has recently been classified as a giant cell-rich histological variant of SFT [22].

Diagnosis of GCA is usually made by pathologic findings after surgical excision and immunohistochemical results. He Y, et al. [23] reported a case that initially diagnosed GCA as vascular malformation, but after complete surgical removal, diagnosis of GCA was confirmed. In general, immunohistochemical features of GCA include positive staining for CD34, CD99, vimentin, and variable Bcl2 and negative staining for CD31, CD68, c-kit/CD117, muscle specific actin, S100, and desmin [21]. Our case was compatible with GCA as immunohistochemical staining showed tumor cells positive for CD34 and Bcl-2, and negative for CD31 and SMA. Though GCA may show similar findings of soft tissue tumors such as giant cell fibroblastoma (GCF), multi-nucleate cell angiohistiocytoma (MCA), vascular malformation (VM), and benign fibrous histiocytoma (BFH) [23], different diagnosis could be made through pathological and immunohistochemical findings. Similarly to GCA, GCF is composed of CD34 positive spindle and stellate shaped cells including multinucleated floret-like giant cells and tumor cell-lined pseudovascular spaces [23]. However, GCF has infiltrative margins and lacks conspicuous vascular pattern characteristic, showing more aggressive lesion than those found in GCA [24]. MCA is a rare benign fibrohistiocytic vascular lesion of the skin, showing vascular proliferation predominantly of capillaries and veins with lymphohistiocyte infiltration as well as positive staining for factor XIIIa [23]. VM can be a consequence of disrupted morphogenesis of arteries, veins, capillaries, lymphatic endothelium alone or a combination of those. Clinical examination of postural movement and a positive puncture test could be used for diagnosis. BFH is a benign mesenchymal neoplasm exhibit fibroblastic and histiocytic characteristics. BFH in the oral cavity presents as a solitary, painless and well-circumscribed nodule [25]. Special stain, such as Masson trichrome stain, can significantly aid in the diagnosis of BFH. In addition, immunohistochemical features of BFH include positive staining for vimentin, actin, CD68, and factor XIIIa [26].

The mTOR pathway, consisting of mTOR complex 1 (mTORC1) and mTOR complex 2 (mTORC2), plays a crucial role in regulating normal cell growth and differentiation [4]. The TSC complex, comprising TSC1, TSC2, and TBC1D7, plays a vital role in tightly controlling the activity of mTORC1, thereby inhibiting excessive cell growth and development of tumors through various mechanisms [27]. When mutations occur in the TSC genes, the regulatory function of mTORC1 is compromised, resulting in the growth of hamartomas, which are characteristic of TSC. These mutations disrupt the normal inhibitory mechanisms of mTORC1 and contribute to the dysregulated cell growth observed in TSC.

Given the rarity of this disease, we report the first case of a TSC patient with giant cell angiofibroma (GCA) in the gingiva. This case contributes to the existing evidence and enhances our understanding of the potential association between TSC and GCA. Further case reports are necessary to clarify the pathogenesis of GCA and provide a deeper understanding of the relationship between TSC and GCA. Although no recurrence was observed in the patient during the 2-year-post surgery follow-up period, it is advisable to maintain regular plaque control and conduct long-term monitoring due to reported cases of gingival enlargement recurrence following gingivectomy [15, 16].

In conclusion, this case report represents the first giant cell angiofibroma in the gingiva of a patient with TSC. We anticipate this report will contribute to a better understanding of this rare disease and the relationship between giant cell angiofibroma and TSC.

## Data Availability

All data generated or analyzed during this study are included in this published article.

## Abbreviations

TSC: Tuberous sclerosis complex
GCA: Giant cell angiofibroma
SFT: Solitary fibrous tumor
mTORC1: Mammalian target of rapamycin complex 1
mTORC2: Mammalian target of rapamycin complex 2
LAM: Lymphangioleiomyomatosis
